# Sports-related injuries and illnesses amongst adolescent athletes in an urban sports medicine practice setting: a one-year prospective study

**DOI:** 10.17159/2078-516X/2025/v37i1a20756

**Published:** 2025-09-15

**Authors:** C van Loggerenberg, DA Ramagole, A Jansen van Rensburg, DC Janse van Rensburg, P Boer

**Affiliations:** 1 Section Sports Medicine, Faculty of Health Sciences, University of Pretoria, South Africa; 2 Department of Human Movement Science, Cape Peninsula University of Technology, Wellington, South Africa

**Keywords:** medical encounters, teenagers, medical centres, epidemiology

## Abstract

**Background:**

Approximately 23 million sports-related injuries occur annually among African adolescents, with limited epidemiological data on sports-related illnesses across the continent.

**Objectives:**

This study highlights the prevalence, nature, and severity of sports injuries and illnesses in adolescent athletes presenting to a South African sports medicine practice.

**Methods:**

A 12-month prospective longitudinal observational study examined adolescent athletes (aged 10–19) presenting to a South African sports medicine practice. At each visit, athletes and their parents/guardians signed consent forms. Data included demographics, sports type, performance level, injuries and illness details and severity according to the 2020 International Olympic Committee (IOC) consensus statement. The primary outcomes included the number (n) and prevalence (%) of injuries and illnesses for each category.

**Results:**

A total of 373 consultations were recorded (202 injuries; 171 illnesses), 61% included male athletes. Team sports athletes accounted for the majority of injuries (64%), while individual sports athletes experienced a higher proportion of illnesses (65%). Lower limb injuries were most common (61%), with joint and muscle sprains (22%) comprising the predominant pathology type. A significant association was found between sex, age, type of sport, and mode of injury onset (p≤0.05). The respiratory system accounted for 54% of all reported illnesses, with infection being the leading cause (97%). For illnesses, significant associations were observed between the type of sport and the affected organ system and aetiology, as well as between sex and aetiology and mode of onset (p≤0.05). Most injuries and illnesses resulted in 1–7 days of time loss.

**Conclusion:**

Male athletes experienced a higher incidence of medical encounters. Injuries were more prevalent in team sports participants, while illnesses were more common in individual athletes. Lower limb injuries, particularly joint and muscle sprains and respiratory infections, cause short-term time loss. This highlights the need for sport-specific injury and illness prevention in adolescent athletes.

Adolescence is a critical period for physical development, during which sports participation plays a key role in promoting health and well-being.^[1]^ However, sports participation also exposes young athletes to a significant risk of injury and illness.^[2–4]^ Globally, around 23 million sports-related injuries occur among adolescents in Africa annually,^[5]^ but the prevalence of illness in adolescent athletes on the continent remains unavailable. At the 2018 Youth Olympic Games (YOG), African countries ranked among the top three regarding injuries and illnesses sustained, highlighting the burden of sports-related health issues in this population.^[6]^

Past research has primarily focused on specific sports within a single season, a particular sporting event or emergency room data.^[2–4,6–11]^ Limited prospective cohort studies investigated adolescent athletes presenting to a sports medicine clinic.^[12,13]^ While previous research has extensively studied injuries in adolescent athletes^[7,8,10,12–18]^, there remains limited evidence on the surveillance of injuries and illnesses within this population.^[2–4,6]^ This is despite existing data showing that both injury and illness have a significant burden on adolescent athletes.^[2,3,19]^ Furthermore, sustaining an injury or illness can negatively impact the adolescent’s sports performance and school attendance.^[19]^

Athlete- and sport-specific factors may contribute to injury and illness patterns among adolescent athletes. Evidence suggests that adolescents sustain a higher proportion of injuries compared to younger paediatric athletes, likely due to increased training demands and physiological growth-related changes.^[12]^ Additionally, injury and illness risks vary by sport type and competition level, with contact sports and higher levels of participation associated with greater injury incidence.^[3,12,13]^ Sports specialisation may further influence injury patterns, as early specialisation in a single sport has been linked to a higher risk of overuse injuries and reduced physical adaptability.^[13,18]^ Furthermore, the severity of an injury or illness directly impacts an athlete’s ability to train and compete, potentially affecting long-term performance and participation in sports.^[2,3]^

A 2023 South African study reported an injury incidence of 34.1/1000 player-hours (95% CI 27.4–40.8) during a single school rugby season, with 77% of injuries resulting in time loss.^[10]^ Similarly, a 2020 South African netball study on adolescent athletes found an injury incidence of 35.1/1000 match hours, with 33% recurrent and 46% gradual-onset injuries over two seasons.^[7]^ In 2015, a Chicago sports clinic reported that 67% of adolescent athlete injuries were gradual-onset, with early sports specialisation increasing the risk (OR 2.25, 95% CI 1.27–3.99; p=0.001).^[13]^ Lower limb injuries were most prevalent across most adolescent sports.^[2,6,7,12,14,15]^

A 6-month prospective study conducted in 2017 at a Norwegian sports academy high school found that 48% of health problems within a single school year were attributed to illnesses, with elite adolescent athletes exhibiting the highest weekly severity scores.^[3]^ Illness incidents in both elite and sub-elite groups surpassed those of acute and gradual-onset injuries. A cross-sectional study of the 2014 USA Youth Olympic Games camp reported 213 illnesses/1000 athletes,^[4]^ a rate significantly higher than the 84 illnesses/1000 athletes reported across all participating teams.^[6]^ These studies, and others, have consistently identified respiratory illnesses as the most common among adolescent athletes.^[2,4,6,11]^

Despite the high number and burden of sports-related injuries and illnesses among adolescents,^[2,4,6,11]^ prospective epidemiological data for this population is limited. Adolescent athletes increasingly engage in organised sports, and understanding the patterns and risks of injury and illness becomes essential for preventing, diagnosing, and managing these conditions. Adolescents are at a critical developmental stage, during which physical demands and vulnerability to injury are heightened, particularly in competitive sports. This study aimed to prospectively record and classify injuries and illnesses among adolescent athletes presenting at a South African sports medicine practice. By providing valuable insights into adolescent athletes’ health, we can inform future strategies for optimising their well-being in sports settings.

## Methods

### Study designs and ethical considerations

A prospective longitudinal observational study analysed sports injury and illness among adolescent athletes who presented to an urban sports medicine practice over twelve months. The Research Ethics Committee of the University of Pretoria granted ethical clearance for this study (432/2022).

### Participant selection

The study included adolescents aged 10 – 19 years^[20]^ who participated in any sporting code and presented to the sports medicine practice between October 2022 and September 2023. Informed consent was obtained from all participants. For participants under 18 years, both parental/guardian consent and participant assent were required. Participants were assessed and treated by one of three sports medicine physicians. The athlete’s demographics and detailed sports participation were noted.

### Data collection

Following consultation, the data on medical encounters (MEs), including injuries and illnesses, were collected and categorised according to the 2020 International Olympic Committee (IOC) consensus statement: methods for recording and reporting epidemiological data on injury and illness in sport.^[21]^ Injury is defined as tissue damage or derangement of normal physical function caused by kinetic energy transfer.^[21]^ Illness is defined as any medical condition unrelated to an injury that affects the athlete’s health.^[21]^

Each clinic visit was recorded and classified as a separate event. Primary demographic data (age and sex), consultation details (date and diagnosis), and participating sports information (specific sport, player position, and level of participation) were collected. Additional data included sport type (team vs. individual sports) and injuries sustained during competition or training. Information on patient management consisted of diagnosis, treatment provided, time loss, and medication prescribed.

Injuries were categorised by anatomical region, body area, tissue and pathology type and mode of onset (acute or gradual-onset).^[ 21]^ Illnesses were categorised by organ system and aetiology^[21]^ and further as acute, progressive, or underlying due to their chronicity or repetitiveness.^[21]^ An athlete was classified as specialised if they participated exclusively in one sporting code and stopped participating in another.^[13,22]^ The level of specialisation was not categorised since the exact duration of single-sport participation was unavailable. Injury and illness severity were based on the number of days lost from participation and grouped into four categories: slight (0 days), minor (1–7 days), moderate (8–28 days), and significant (>28 days).^[7,21]^

### Statistical analysis

All data were analysed using SAS statistical software (V.9.4; Cary, North Carolina, USA) via counts and percentages of illnesses and injuries. Injuries and illnesses were analysed according to sport type (team or individual sports), highly specialised (if participated in a single sport), performance level (school/club, provincial, national), and severity. Classification variables were coded according to the IOC categories.^[21]^ Injuries were classified by anatomical region, pathology type and mode of onset (acute, gradual-onset). Illnesses were classified by organ system, aetiology, and mode of onset (acute, progressive, underlying). Athletes who were ill or injured more than once during the study period were entered as unique entries. Chi-square and Fisher’s exact tests were used to assess significant associations between categorical data, with p≤0.05 indicating significance.

## Results

### Demographics

#### Numbers and frequencies

During the twelve months, 373 MEs were recorded (202 injuries; 171 illnesses). Male athletes had the most MEs, comprising 68% of injuries and 53% of illness cases. Most visits were from athletes in the 13–16 age group (62%). Team sports participants accounted for 64% of all reported injuries, while those in individual sports accounted for 65% of illnesses. Most MEs resulted from school/club performance levels, constituting 61% of injuries and 53% of illnesses. Adolescent athletes not highly specialised (57%) had more injuries compared to highly specialised (43%) athletes, whilst for illnesses, the distribution was even (50%). Injury severity was primarily classified as minor (41%) and moderate (30%). Most illnesses were of minor severity (65%), followed by slight severity (33%) (Table 1).

#### Association

A significant association was found between age and sex in the injury group (χ^2^=6.37, p≤0.05), with a higher percentage of males in the middle and older age groups compared to females. The illness group had no significant associations between age and sex (χ^2^=4.01, p≥0.05). In the illness group, a significant association between team/individual sport and sex was identified (χ^2^=8.92, p≤0.05), where a higher percentage of males were ill in team sports, while females were more frequently affected by illness in individual sports. No statistically significant association between sex and injury severity (χ^2^=6.37, p≥0.05), with a significant association between sex and illness severity identified (p≤0.05), with females experiencing a higher proportion of slight illnesses and males experiencing more minor illnesses (Table 1).

### Injuries

#### Numbers and frequencies

Lower limb injuries accounted for 61% of all injuries. (Table 2) Knee (n=40, 20%), ankle (n=27, 13%), and shoulder (n=25, 12%) injuries comprised two-thirds of all injuries. The shoulder (55%) was the most common area injured in the upper limb (Table 2).

Lower limb injuries accounted for the most injuries in all major sports types, with rugby contributing the highest number of injuries in all anatomical regions except trunk and back injuries, which were most frequent in cricket.

No head, neck or face injuries were reported in males participating in athletics and wrestling, or in females participating in netball (Supplementary table 1). Gradual-onset injuries did not affect the head, neck, and face region. In contrast, the back and trunk region presented more gradual-onset injuries than acute injuries (n=13 vs n=4) (Supplementary Table 2).

Of the recorded injuries, 112 (56%) were classified as acute and 87 (44%) as gradual-onset. Acute injuries in team sports represented 65% of all team sports injuries. In comparison, 58% of injuries in individual sports were gradual-onset. Cricket was the only team sport in which the percentage of gradual-onset injuries exceeded acute injuries (23% vs 7%, respectively). Conversely, among the 14 individual sports, only athletics had more acute injuries than gradual-onset injuries (n=18 vs n=15, respectively) (Supplementary Table 2).

**Fig. 1:**
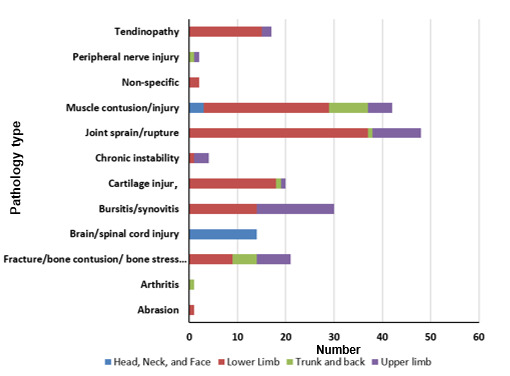
Injury pathology type categorised by anatomical location (n=202)

Joint sprain and tendon rupture pathology type accounted for 24% of all injuries, followed by muscle injuries (21%). In team sports, joint sprains and tendon ruptures (30%) were the most common pathologies, while muscle contusions were the leading pathology among individual sport athletes (21%) (Table 2). Figure 1 illustrates the distribution of pathology types by anatomical locations. The lower limb was involved in most joint sprains, muscle injuries, tendinopathies and cartilage injuries, while bursitis was nearly equally divided between the upper and lower limbs. Chronic instability primarily affected the upper limb. All four anatomical locations showed muscle contusions/injuries.

#### Association

There was a significant association between teams/individuals and mode of onset (p≤0.05), with team athletes showing more acute injuries and individual athletes representing more gradual onset injuries (Table 2).

### Illness

#### Numbers and frequencies

**Fig. 2:**
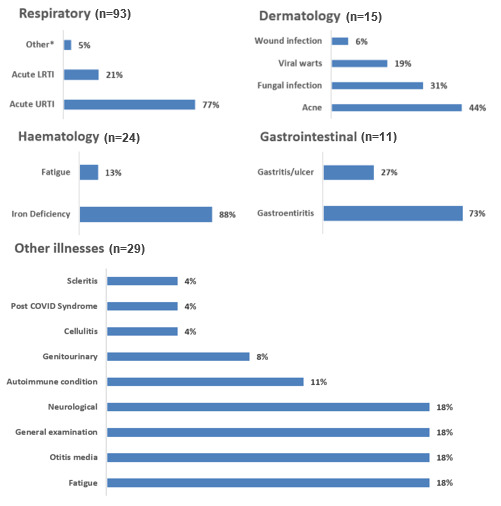
Percentage distribution of diagnoses for each organ system (n=172). ^*^Allergic rhinitis, chronic sinusitis, chronic nasal obstruction, respiratory hyperactivity, and COVID.

The organ system most affected was the respiratory system (54%), which involved team (68%) and individual (47%) athletes. The respiratory system accounted for the highest percentage of illnesses in team and individual sports, with haematological illness occurring more frequently in individual sports. Athletes in individual sports presented with 23 out of the 24 (96%) haematological illnesses, with 87% diagnosed with iron deficiency (Figure 2). Infection caused most illnesses (68%), with 78% due to respiratory infections. Metabolic and nutritional aetiology affected 20% of female athletes and was only documented in athletes participating in individual sports. Metabolic and nutritional factors were more associated with individual athletes and females, whereas infection was reported more in team sports and males. Acute illness occurred in 72% of all cases (Table 3).

Three-quarters of all respiratory illnesses were acute upper respiratory tract infections (URTIs). Dermatological conditions included acne (44%) and fungal infection (31%). Gastroenteritis (73%) was the leading cause of gastrointestinal illness. Other consultations included diagnoses such as autoimmune and neurological illnesses, as well as general medical conditions (Figure 2).

Regarding illness distribution by sport, athletics, rugby, and wrestling were the three most frequent male sports presenting with an illness. Among females, the top three sports with the most frequent illnesses were athletics, tennis and netball. Athletics accounted for the highest number of athletes presenting with a haematological issue, while wrestling had the highest number of dermatological presentations (Supplementary Table 3).

#### Association

A significant association was found between sports type (team vs individual) and the organ system involved (p≤0.05), where team athletes demonstrated more respiratory illnesses. A significant association between aetiology and sports type (p≤0.05), with individual sports representing all metabolic/nutritional illnesses and between aetiology and sex (χ^2^=9.33, p≤0.05), with females representing more of the metabolic/nutritional and other aetiologies. Additionally, a significant association between mode of onset and sex was demonstrated (p≤0.05), with progressive and underlying illness occurring more frequently in females compared to males.

## Discussion

This study aimed to prospectively record and classify injuries and illnesses in adolescent athletes at a South African sports medicine practice over 12 months. It represents a first-of-its-kind study in South Africa focusing on injuries and illnesses in adolescent athletes. The key findings were that male athletes accounted for 61% of all MEs, with team sports having the most injuries (64%) and individual sports having the most illnesses (65%). These results align with previous research, which found that injuries resulted in more MEs than illnesses.^[2–4,6]^

Fifty-two percent of all visits resulted in minor severity, with 11% of injuries and 33% of illnesses resulting in no time loss. In a 25-week prospective cohort study involving seventeen adolescent rugby players from the English Super League Academy squad, 65% of those who experienced an illness reported no loss of training time or reduction in training volume. ^[11]^ In a prospective cohort study among Swedish adolescent floorball players, 51% of all illnesses resulted in no time loss.^[9]^ Both these studies followed the adolescent athletes on a weekly basis,^[9,11]^ compared to our study, which only included patients presenting with MEs, which could account for the lower no-time loss visits. Further, a time loss of 1–7 days, as the most frequent severity of MEs, could be attributed to athletes seeking medical attention early for conditions requiring further investigation.

### Injuries

In this study, adolescent males sustained 68% of injuries compared to previous studies that reported 60% of injuries in adolescent males.^[3,16]^ In a prospective study on elite distance runners in the United Kingdom, 45% of injuries occurred in males.^[2]^ A prospective cohort study at the 2018 YOG reported 619 injuries, 52% occurring in male adolescent athletes.^[6]^ The distribution of injuries by sex appears to be influenced by sports type, study design and the specific population studied. Our study showed a significant association between sex and age group (χ2=6.37, p≤0.05) but not between sex and other demographic variables. In a study conducted in Luxembourg over 24 months, following adolescents attending a sports school, age was found to be a protective factor, with the older adolescents sustaining fewer injuries.^[14]^ However, this study does not provide information pertaining to the age categories and no breakdown of age by sex, and thus it is difficult to compare our results to this similar prospective cohort study.

We found that lower limb injuries (61% of all injuries) were the most common anatomical region involved in both male (55%) and female (73%) adolescent athletes, as reported in other adolescent studies.^[2,6,7,12,14,15,17]^ The adolescent sports code could influence which anatomical area is most affected by injury. In our study, rugby accounted for the highest percentage of injuries across all regions except the trunk and back, with lower limb injuries most prevalent. This contradicts findings from a prospective South African school rugby study, which followed a team over one season, and reported the head, neck, and spine as the most frequently injured anatomical areas.^[10]^ Similarly, a 2020 prospective study on South African netball players over two seasons reported that 13% of injuries involved the head, neck and face region.^[7]^ In contrast, our study observed no injuries in this region in netball players (Supplementary Table 1). An essential difference between our research and these studies is that they focus on a single sport with higher participant numbers in each code. Our study found that cricket accounted for the most trunk and back injuries. These findings indicate the importance of further research in sport-specific adolescent injuries in South Africa to aid in focused prevention strategies.

At the 2018 multi-day YOG joint, muscle and bone injuries were reported to be the predominant pathology type. Ligament strain or rupture was associated with 22% of all injuries.^[6]^ These findings are similar to those of the current study, where joint and tendon sprains and ruptures (24%) and muscle injuries (21%) were commonly reported. Another South African prospective study focusing on netball players reported that ligament injuries (36%) were the most frequent.^[7]^

In our study, a significant association was observed between sport type (team vs individual) and acute vs gradual-onset injuries (p≤0.05), indicating that sport type influences the mode of onset of injury, with individual sport having a higher percentage of gradual-onset injuries (58%), and team sports more acute injuries (64%), despite team sport (n=45) and individual sport (n=42) having an almost equal distribution. In contrast, a prospective cohort study of adolescent athletes in a public sports school in Luxembourg found no association between team/individual sport and mode of onset of injury (p =0.25). In this cohort, team sport had a higher competition rate and individual sports a higher training load. ^[14]^ A retrospective chart review of gradual-onset injuries from a paediatric sports medicine clinic in Chicago over five years identified track and field/cross country (18%), baseball (15%) and soccer (14%) as the most common sports with gradual-onset injuries.^[12]^ In comparison, our study found cricket (23%), athletics (17%), and rugby (13%) to be the leading contributors to gradual-onset injuries These differences in sport types between European and American settings, compared to a South African context, highlight the need for larger, sport-specific studies in South Africa that focus on team and individual sports and incorporate risk factors, such as training and competition loads, in the development of acute and gradual-onset injuries.

### Illness

Over 12 months, 171 illnesses (46% of all MEs) were reported, 53% males and 47% females. Our results are comparable to other prospective cohort studies reporting injury and illness in adolescent athletes. A Norwegian study conducted over 6 months at a sporting school with multiple sporting codes reported that 48% of MEs were illness-related^[3]^; in single-sport code studies, the illness-related MEs ranged from 41–45%.^[2,9]^ A prospective study of adolescent rugby players in the English Super League Academy squad found that 65% reported at least one illness over 25 weeks.^[11]^

A slight difference was observed in the percentage of male and female athletes presenting with an illness (53% vs 47%). Female and male football players in Sweden also presented with a similar illness prevalence (13% and 11%, respectively).^[9]^ In the 2018 YOG, females had a significantly higher risk ratio to present with an illness (RR=1.55, 95% CI 1.24 to 1.93).^[6]^ Research on a larger cohort of adolescent athletes is needed to confirm whether there is a difference in male and female athletes’ illness presentation and severity.

The respiratory system is the most frequently recorded primary organ system affected in adolescent athletes across various studies. ^[2,4,6,9,11]^ The digestive and dermatological systems are the second and third most common. ^[2,6,11]^ In our study, the respiratory system was the primary system most involved, followed by the haematological system, which predominantly involved athletes from individual sports.

A significant association was found between team/individual sports type and organ system involvement (p≤0.05). Sixty-eight percent of all illnesses were due to an infection, comparable to other studies on illness in adolescent athletes. ^[2–4,6,9,11]^ The significant association between aetiology and team/individual sports (p≤0.05) in our research has not been noted before, with more specific detail on metabolic factors affecting adolescents participating in individual sports, particularly females, highlighted explicitly in our research, which warrants further evaluation.

In our study, athletes participating in individual sports had more illnesses than those in team sports (65% vs 35%). It aligns with reports from the YOG, where individual sports such as golf had the highest illness incidence (20.3 illnesses/100 athletes), followed by triathlon (15.6 illnesses/100 athletes).^[6]^ In a prospective cohort study of Norwegian sports schools, endurance athletes had a higher prevalence of illness than those in technical (p=0.035) and team sports (p=0.002).^[3]^ The training load associated with endurance and individual sports could contribute to these findings. However, further research is necessary to understand specific risk factors for illness.

Our study found multiple significant associations within the illness group between sex and sport type (χ^2^=8.92, p≤0.05), illness severity (p≤0.05), aetiology (χ^2^=9.33, p≤0.05), and mode of onset (p≤0.05). These findings, and the limited representation of some of these findings in existing research, underscore the need for illness-specific research in adolescent sports using larger cohorts. Notably, haematological illness only affects individual sports and occurs more frequently in females than males. Given its under-representation in current research, this area warrants further investigation to inform targeted educational initiatives for athletes and coaches.

### Strengths and limitations

This study, which reports on adolescent athletes visiting a South African sports medicine practice, offers several strengths, particularly its focus on illness and injury patterns within a unique and understudied population. The prospective nature of this study allowed for detailed and systematic data collection over 12 months. The study provides valuable insights specific to adolescent athletes with distinct physiological and developmental characteristics, highlighting the prevalence of lower limb injuries and respiratory illnesses, in alignment with existing literature. It further emphasises the importance of monitoring health issues in a high-performance youth setting, while providing a foundation for future research and strategies to optimise the wellbeing of adolescent athletes. This study’s limitations include the small sample size and single-centre data collection. Sports medicine practices nationwide may have differing adolescent athlete populations, affecting the types of injuries and illnesses observed. Although this study did not collect data on potential risk factors, incorporating a patient questionnaire to assess aspects such as training load, period of specialisation, injury and illness history, and other potential risk factors will offer valuable insights for sports medicine clinicians, health professionals and those working in the field of adolescent sport. The body of knowledge could be further enhanced with a multicentre prospective study incorporating a patient questionnaire to assess risk factors in specific sports.

## Conclusion

This study highlights the prevalence, nature, and severity of sports injuries and illnesses in adolescent athletes presenting to a sports practice in South Africa. Male athletes had more injuries and illnesses. Injuries were higher in team sports and illnesses in individual sports, highlighting the need for a different focus in specific sports. The majority of adolescents were in the 13–16-year age group. Lower limb injuries, injuries from joint sprains and strains, respiratory illness, and infectious pathology, which cause short-term time loss, need targeted interventions. This research also indicates the need for further research on sports that are underrepresented in research focusing on the Northern hemisphere. This research also shows the significant association between team/individual sport, sex and illness categorisation, highlighting the need for further research on the impact of illness and associated risk factors in adolescent athletes.

## Supplementary Information



## Figures and Tables

**Table 1:** The characteristics of injury and illness visits to the sports medicine clinic (n (%))

	All MEs			Injury			Illness		

	Male	Female	Total	Male	Female	Total	Male	Female	Total

**Total visits**	229 (61)	144 (39)	373 (100)	138 (68)	64 (32)	202 (100)	91 (53)	80 (47)	171 (100)

**Age groups**				*	*				
10 to 12 years	25 (11)	21 (15)	46 (12)	11 (8)	13 (20)	24 (12)	14 (15)	8 (10)	22 (13)
13 to 16 years	148 (65)	82 (57)	230 (62)	89 (64)	36 (56)	125 (62)	59 (65)	46 (58)	105 (61)
17 to 19 years	56 (24)	41 (28)	97 (26)	38 (28)	15 (23)	53 (26)	18 (20)	26 (33)	44 (26)

**Sports Type**							*	*	
Team sport	134 (58)	54 (38)	188 (50)	93 (67)	36 (56)	129 (64)	40 (45)	19 (23)	59 (35)
Individual sport	96 (42)	89 (62)	185 (50)	45 (33)	28 (44)	73 (36)	51 (55)	61 (77)	112 (65)

**Highly specialised**									
Yes	100 (44)	72 (50)	172 (46)	61 (44)	25 (39)	86 (43)	39 (43)	47 (59)	86 (50)
No	129 (56)	72 (50)	201 (54)	77 (56)	39 (61)	116 (57)	52 (57)	33 (41)	85 (50)

**Performance Level**									
School/Club	138 (60)	76 (53)	214 (57)	89 (64)	34 (53)	123 (61)	49 (54)	42 (53)	91 (53)
Provincial	60 (26)	38 (26)	98 (26)	33 (24)	21 (33)	54 (27)	27 (30)	17 (21)	44 (26)
National	31 (14)	30 (21)	61 (16)	16 (12)	9 (14)	25 (12)	15 (16)	21 (26)	36 (21)

**Severity**							#	#	
Slight (0 days)	33 (14)	45 (31)	78 (21)	10 (7)	12 (19)	22 (11)	23 (25)	33 (41)	56 (33)
Minor (1–7 days)	127 (55)	68 (47)	195 (52)	60 (44)	23 (36)	83 (41)	67 (74)	45 (56)	112 (65)
Moderate (8–28 days)	45 (20)	18 (13)	63 (17)	44 (32)	17 (27)	61 (30)	1 (1)	1 (1)	2 (1)
Major (>28 days)	24 (11)	13 (9)	37 (10)	24 (17)	12 (19)	36 (18)	0 (0)	1 (1)	1 (1)

N, number %; percentage MEs Medical Encounters; Percentages are calculated for injury and illness groups separately.

* Significant association between categories as per Chi-square analysis (p ≤ 0.05);

# Significant association between categories as per Fisher’s exact test (p ≤ 0.05).

**Table 2:** Anatomical location, pathology type, and mode of onset of all injuries, team vs. individual athletes, by sex (male, female) (n (%))

Anatomical region	Total n = 202	Team n = 129	Individual n = 73	Male n = 138	Female n = 64

**Lower Limb**	**123 (61)**	**75 (58)**	**48 (66)**	**76 (55)**	**47 (73)**
Knee	40 (20)	26 (20)	14 (19)	27 (20)	12 (19)
Ankle	27 (13)	17 (13)	10 (14)	13 (9)	14 (22)
Thigh	18 (9)	9 (7)	9 (12)	14 (10)	4 (6)
Foot	16 (8)	9 (7)	7 (10)	8 (6)	8 (13)
5Hip/Groin	16 (8)	11 (9)	5 (7)	11 (8)	5 (8)
Leg	5 (3)	3 (2)	2 (3)	2 (2)	3 (5)

**Upper limb**	**45 (22)**	**30 (23)**	**15 (21)**	**35 (25)**	**10 (16)**
Shoulder	25 (12)	17 (13)	8 (11)	23 (17)	2 (3)
Hand	9 (4)	5 (4)	4 (5)	7 (5)	2 (3)
Other (Elbow, forearm, wrist, upper arm)	12 (6)	8 (6)	4 (5)	6 (4)	6 (9)

**Head, neck, and face**	**17 (8)**	**14 (11)**	**3 (4)**	**15 (11)**	**2 (3)**
Head and face	14 (7)	11 (9)	3 (4)	12 (9)	2 (3)
Neck	3 (1)	3 (2)	0 (0)	3 (2)	0 (0)

**Trunk and back**	**17 (8)**	**10 (8)**	**7 (10)**	**12 (9)**	**5 (8)**
Lumbosacral	15 (7)	9 (7)	6 (8)	11 (8)	4 (6)
Other (Abdomen, thoracic spine)	2 (1)	1 (1)	1 (1)	1 (1)	1 (2)

**Pathology Type**					

Joint sprain/rupture	48 (24)	39 (30)	9 (12)	31 (22)	17 (27)
Muscle contusion/injury	42 (21)	27 (21)	15 (21)	29 (21)	13 (20)
Bursitis/synovitis	30 (15)	19 (15)	11 (15)	25 (18)	5 (8)
Fracture/bone contusion/bone stress injury/Physis injury	21 (10)	10 (8)	11 (15)	14 (10)	7 (11)
Cartilage injury	20 (10)	7 (5)	13 (18)	13 (9)	7 (11)
Tendinopathy	17 (8)	9 (7)	8 (11)	9 (7)	8 (13)
Brain/spinal cord injury	14 (7)	11 (9)	3 (4)	12 (9)	2 (3)
Other (abrasion/arthritis/chronic instability/non-specific/peripheral nerve)	10 (5)	7 (5)	3 (4)	6 (4)	4 (6)

**Mode of onset** *		#	#		
Acute injury	112 (56)	81 (64)	31 (42)	78 (58)	34 (53)
Gradual-onset injury	87 (44)	45 (36)	42 (58)	57 (42)	30 (47)

n, number; %, percentage.

* Mode of onset has missing data for three visits.

# Significant association between categories as per Fisher’s exact test (p≤0.05).

**Table 3:** Illness by organ system, aetiology, and mode of onset for all athletes, team vs. individual athletes, by sex (male, female) (n (%))

	Total n = 171	Team n = 59	Individual n = 112	Male n = 91	Female n = 80

**Organ System**		#	#		
Respiratory	93 (54)	40 (68)	53 (47)	53 (58)	40 (50)
Dermatological	15 (9)	5 (8)	10 (9)	9 (10)	6 (8)
Haematological	24 (14)	1 (2)	23 (21)	9 (10)	15 (19)
Gastrointestinal	11 (6)	5 (8)	6 (5)	9 (10)	2 (3)
Other&	28 (16)	8 (14)	20 (18)	11 (12)	17 (21)

**Aetiology**		#	#	*	*
Infection	116 (68)	50 (85)	66 (59)	71 (78)	45 (56)
Metabolic/Nutritional	26 (15)	0 (0)	26 (23)	10 (11)	16 (20)
Other+	29 (17)	9 (15)	20 (18)	10 (11)	19 (24)

**Mode of onset**				#	#
Acute illness	123 (72)	47 (80)	76 (68)	73 (80)	50 (63)
Progressive illness	39 (23)	11 (19)	28 (25)	17 (19)	22 (28)
Underlying illness	9 (5)	1 (2)	8 (7)	1 (1)	8 (10)

n, number; %, percentage.

* Significant association between categories as per Chi-square analysis (p ≤ 0.05);

# Significant association between categories as per Fisher’s exact test (p ≤ 0.05).

& Other organ systems: cardiovascular, gastrointestinal, genitourinary, musculoskeletal, neurological, non-specified, ophthalmological, otological, psychiatric/psychological.

+ Other aetiology: allergic, degenerative/chronic condition, development anomaly, environmental exercise-related, environmental non-exercise, immunological/inflammatory, not-specified.
