# Supplementary material for: Sports-related injuries and illnesses amongst adolescent athletes in an urban sports medicine practice setting: a one-year prospective study
Source: S Afr J Sports Med. 2025 Sep 15;37(1):v37i1a20756. doi: 10.17159/2078-516X/2025/v37i1a20756 (PMC12520735; doi:10.17159/2078-516X/2025/v37i1a20756)
Supplement: Supplementary file 1 [file 2078-516X-37-v37i1a20756-s001.pdf]

Sports-related injuries and illnesses amongst adolescent athletes in an urban sports medicine practice setting: a one-year prospective study

Supplementary Table 1: Anatomical region and severity of injury in the most frequently injured sports for males and females (n (%))

		**Anatomical region**				**Severity**			
Gender	Sport	Head, neck, and face	Upper Limb	Trunk and back	Lower Limb	0 Days	1-7 Days	8-28 Days	>28 Days
**Male**	Rugby (n=51)	11 (8)	16 (12)	1 (1)	23 (17)	5 (4)	23 (17)	16 (12)	7 (5)
	Cricket (n=29)	0 (0)	6 (4)	6 (4)	17 (12)	2 (1)	15 (11)	11 (8)	1 (1)
	Athletics (n=15)	1 (1)	2 (1)	2 (1)	10 (7)	1 (1)	5 (4)	6 (4)	3 (2)
	Wrestling (n=10)	0 (0)	2 (1)	1 (1)	7 (5)	1 (1)	4 (3)	2 (1)	3 (2)
	All Sports (n=138)	15 (11)	35 (25)	12 (9)	76 (55)	10 (7)	60 (44)	44 (32)	24 (17)
**Female**	Athletics (n=19)	1 (2)	1 (2)	3 (5)	14 (22)	4 (6)	6 (9)	6 (9)	3 (5)
	Netball (n=16)	0 (0)	3 (5)	0 (0)	13 (20)	3 (5)	4 (6)	5 (8)	4 (6)
	Hockey (n=15)	1 (2)	2 (3)	2 (3)	10 (16)	3 (5)	7 (11)	4 (6)	1 (2)
	All Sports (n=64)	2 (3)	10 (16)	5 (8)	47 (73)	12 (19)	23 (36)	17 (27)	12 (19)

n: number %: percentage

Supplementary Table 2: Mode of onset for anatomical location and sporting codes (n (%))

	**Total (n=199)**	**Acute (n=112)**	**Gradual-onset (n=87)**
**Anatomical region**			
Lower Limb	120 (60)	61 (55)	59 (68)
Upper limb	45 (22)	30 (27)	15 (17)
Head, neck, and face	17 (9)	17 (15)	0 (0)
Trunk and back	17 (9)	4 (4)	13 (15)
**Sporting code**			
**Team sports**	**126 (63)**	**81 (72)**	**45 (51)**
Rugby	51 (26)	40 (36)	11 (13)
Cricket	28 (14)	8 (7)	20 (23)
Hockey	23 (12)	16 (14)	7 (8)
Netball	16 (8)	10 (9)	6 (7)
Soccer	4 (2)	3 (3)	1 (1)
Basketball	2 (1)	2 (2)	0 (0)
Softball	2 (1)	2 (2)	0 (0)
**Individual sports**	**73 (37)**	**31 (28)**	**42 (49)**
Athletics	33 (17)	18 (16)	15 (17)
Wrestling	10 (5)	5 (4)	5 (6)
Cross Country	5 (3)	1 (1)	4 (5)
Cycling	5 (3)	3 (3)	2 (2)
Golf	4 (2)	1 (1)	3 (4)
Other*	16 (8)	3 (3)	13 (15)

n, number; %, percentage

**Other: Dance (3), Kickboxing (3), Swimming (3), Tennis (2), Acrobatics (1), Gymnastics (1), Motorcross (1), Rock Climbing (1), and Triathlon (1)*

Supplementary Table 2: Mode of onset for anatomical location and sporting codes (n (%))

		Organ System				Severity			
**Gender**	**Sport**	**Respiratory**	**Haematological**	**Dermatological**	**Other***	**0 Days**	**1-7 Days**	**8-28 Days**	**>28 Days**
**Male**	Athletics (n=8)	3 (3)	3 (3)	1 (1)	1 (1)	5 (5)	3 (3)	0 (0)	0 (0)
	Rugby (n=24)	15 (17)	0 (0)	1 (1)	8 (9)	5 (5)	18 (20)	1 (1)	0 (0)
	Wrestling (n=29)	19 (22)	1 (1)	4 (5)	5 (6)	3 (3)	26 (29)	0 (0)	0 (0)
	All Sports (n=91)	53 (58)	9 (10)	9 (10)	20 (22)	23 (25)	67 (74)	1 (1)	0 (0)
**Female**	Athletics (n=28)	11 (14)	8 (10)	2 (3)	8 (10)	17 (21)	10 (13)	1 (1)	1 (1)
	Tennis (n=12)	7 (9)	0 (0)	1 (1)	4 (5)	2 (3)	10 (13)	0 (0)	0 (0)
	Netball (n=9)	8 (10)	0 (0)	0 (0)	1 (1)	1 (1)	8 (10)	0 (0)	0 (0)
	All Sports (n=80)	40 (50)	15 (19)	6 (8)	19 (24)	33 (41)	45 (56)	1 (1)	1 (1)

n, number; %, percentage

* Other organ systems: cardiovascular, gastrointestinal, genitourinary, musculoskeletal, neurological, not specified, ophthalmological, otological, psychiatric/psychological.
